# Imaging Diagnosis of Interstitial Pneumonia with Emphysema (Combined Pulmonary Fibrosis and Emphysema)

**DOI:** 10.1155/2012/816541

**Published:** 2012-02-09

**Authors:** Fumikazu Sakai, Junya Tominaga, Akiko Kaga, Yutaka Usui, Minoru Kanazawa, Takashi Ogura, Noriyo Yanagawa, Tamiko Takemura

**Affiliations:** ^1^Department of Diagnostic Radiology, Saitama International Medical Center, Saitama Medical University, Hidaka, Saitama 350-1298, Japan; ^2^Department of Respiratory Medicine, Saitama Medical University, Moroyama-Machi, Saitama 350-0040, Japan; ^3^Department of Respiratory Medicine, Kanagawa Prefectural Cardiovascular and Respiratory Disease Center, Yokohama 236-0051, Japan; ^4^Department of Radiology, Tsukuba Memorial Hospital, Tsukuba 300-2622, Japan; ^5^Department of Pathology, Japanese Red Cross Medical Center, Tokyo 150-8935, Japan

## Abstract

Based on clinical and radiological findings, Cottin defined combined pulmonary fibrosis and emphysema (CPFE) as pulmonary emphysema in the upper lungs and interstitial pneumonia in the lower lungs with various radiological patterns. Pathologic findings of CPFE probably corresponded with diffuse interstitial pneumonia with pulmonary emphysema, emphysema with fibrosis, and the combination of both. We described reported radiological findings of CPFE.

## 1. Introduction

Interstitial pneumonia (IP) with pulmonary emphysema has long been a topic of controversy because we have not decided if the disease is simple coincidence of IP and emphysema or IP and emphysema may be caused by common etiology. In 1990, Wiggins et al. [1] presented 8 cases in the first report in the English literature. In 2005, Cottin et al. [2] described combined pulmonary fibrosis and emphysema (CPFE) based on clinical and radiological findings and noted some characteristic clinical features. Description of the pathological mechanisms in such cases is very limited.

 Defined by clinical and imaging findings, CPFE is one of clinicoradiological syndromes and probably includes several kinds of pulmonary fibrosis, so its clinical course and prognosis various [3]. It is unclear how CPFE should be categorized under interstitial pneumonia to ensure the integrity of idiopathic interstitial pneumonia (IIP) classification. We herein review and summarize the imaging features of CPFE.

## 2. Smoking and Interstitial Pneumonia

Many pulmonary diseases are strongly associated with smoking-pulmonary emphysema, lung cancer, and some specific interstitial diseases, such as pulmonary Langerhans cell histiocytosis (LCH), desquamative interstitial pneumonia (DIP), and respiratory bronchiolitis interstitial lung disease (RBILD) [4–7].

Coexistence of pathologic findings of LCH, DIP, or RBILD in the same patient with smoking habit has been reported, and these three disease entities were sometimes categorized as smoking-related interstitial lung disease (SRILD) in narrow definition [7, 8]. However, in a broad sense of term, SRILD may include pulmonary emphysema, IPF, and CPFE.

 Smoking and/or dust inhalation are also important factors in the development of idiopathic interstitial pneumonias [9], and most patients with idiopathic pulmonary fibrosis (IPF) have a history of smoking [10]. Overt pulmonary emphysema complicates IPF in approximately 30% of patients [11]. Iwai et al. calculated an odds ratio of 3.4 relating IPF to smoking or dust inhalation [12]. A greater prevalence of nonspecific interstitial pneumonia (NSIP) is also reported in smokers [13], with DIP and RBILD generally confined to current smokers [8, 14–16].

Smoking has been closely related to DIP, RBILD, and LCH [8, 17], and features of all three are reported in pathologic specimens of one individual [16, 17]. These 3 diseases and IPF are also generalized as smoking-related interstitial lung disease (SRILD) [4, 5, 18].

Based on clinical and imaging findings, Cottin et al. also rediscussed CPFE as pulmonary emphysema, defined as low-attenuation areas surrounded by normal lung with thin or no wall and/or multiple bullae predominantly in upper lungs, and interstitial pneumonia in the lower lungs with imaging patterns of various IPs, including usual interstitial pneumonia (UIP), NSIP, RBILD, cryptogenic organizing pneumonia (OP), and others [8].

Cottin's article reported characteristic clinical features including poor prognosis, frequently complicated by pulmonary hypertension [19, 20], preserved forced expiratory volume in one second (FEV 1.0), and severely impaired diffusion capacity [2, 21]. Mejía et al. also reported decreased survival of patients with IPF and emphysema with pulmonary hypertension [10]. However, we believe that the prognosis of interstitial pneumonia is better than that of IPF/UIP [22], despite a high prevalence of lung cancer reported in cases of CPFE [23, 24] and even IPF/UIP [25].

 In a mass screening of smokers in the United States, the COPD Gene Study Group reported interstitial shadow or radiologically overt IP on high-resolution computed tomography (HRCT) in approximately 8% of 2500 subjects with chronic obstructive pulmonary disease (COPD) [26, 27]. They categorized interstitial changes on HRCT into following four types: centrilobular, subpleural, mixed abnormal opacity, or radiologically overt IP. On pulmonary function tests, total lung capacity was increased in subjects with centrilobular or mixed opacity but not in those with subpleural abnormal opacity and overt IP. These results suggested a correlation between interstitial lung change and smoking.

 Pulmonary injury may cause pulmonary destruction in emphysema or abnormal repair processes in fibrosis. Smoking can cause simultaneous pulmonary emphysema and fibrosis. Bates et al. [28, 29] described changes in the lung matrix as a common pathway of destructive change and fibrosis. However, CPFE may not be a single disease and may include heterogeneous diseases [3].

## 3. Pathologic Features of CPFE

Pathologic features of SRILD in narrow definition (DIP, RBILD, and LCH) have been described [7, 30–32]. Desquamative interstitial pneumonia (DIP) shows accumulation of alveolar macrophages within the alveolar lumen and mild fibrotic thickening of the alveolar septa, but advanced fibrosis is uncommon. RBILD shows accumulation of alveolar macrophages laden with brown pigment within the respiratory bronchioles. Langerhans cell histiocytosis results when Langerhans histiocytes and eosinophils form granulomas in the upper lung, cavitary nodules, and cysts by the necrosis of granulomas.

Although CPFE is closely related to smoking, its pathologic features differ. In noncancerous portions of lung specimens obtained by lobectomy for bronchogenic carcinoma, Kawabata et al. [33] so frequently observed relatively thick-walled cysts that they designated the finding as air space enlargement with fibrosis (AEF). They depicted AEF related to smoking index and possibly induced by smoking. Katzenstein et al. [34] found similar hyalinizing collagenous fibrotic change of the alveolar septa along the emphysematous walls and respiratory bronchioles in the lungs of smokers; change that they designated occult fibrosis of smokers or smoking-related interstitial fibrosis.

Wright et al. [35] described 2 types of smoking-related interstitial lung disease-diffuse form, which includes overlapping pulmonary emphysema and IP, such as UIP or NSIP, and localized form, which includes emphysema with fibrosis, AEF, or occult fibrosis of smokers. Although pulmonary emphysema is defined as air space without fibrosis, Wright described frequently varying degrees of pulmonary fibrosis in the alveolar septa around the emphysema wall. Radiological evaluation aids differentiation of diffuse and localized forms. Fibrotic change is also identified along bronchioles in addition to alveolar septa around pulmonary emphysema [31].

However, pathologic features of CPFE have not been fully studied; thus further investigation is mandatory to show pathologic characteristics of CPFE.

## 4. Imaging Features of CPFE

**Figure 1:**
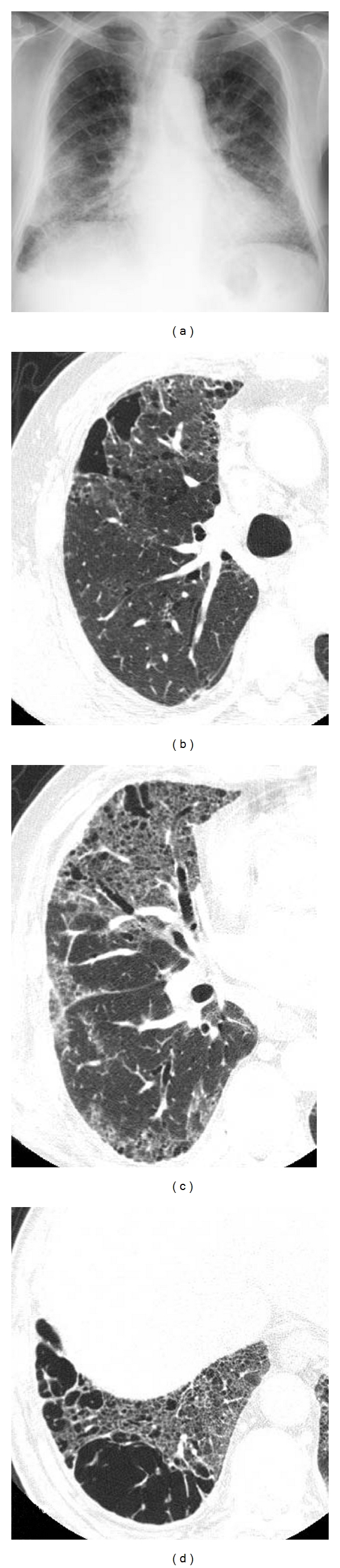
Diffuse interstitial pneumonia (nonspecific interstitial pneumonia (NSIP) pattern) with emphysema. (a) Chest X-ray shows bibasilar ground glass and reticular opacity. Volumes of bilateral lungs are almost normal. (b) High-resolution computed tomography (HRCT) of upper lung shows centrilobular pulmonary emphysema. (c), (d) HRCT of lower lung shows ground glass opacity along the bronchovascular bundle including cysts with varying size. Distribution of abnormal opacity mimics NSIP.

**Figure 2:**
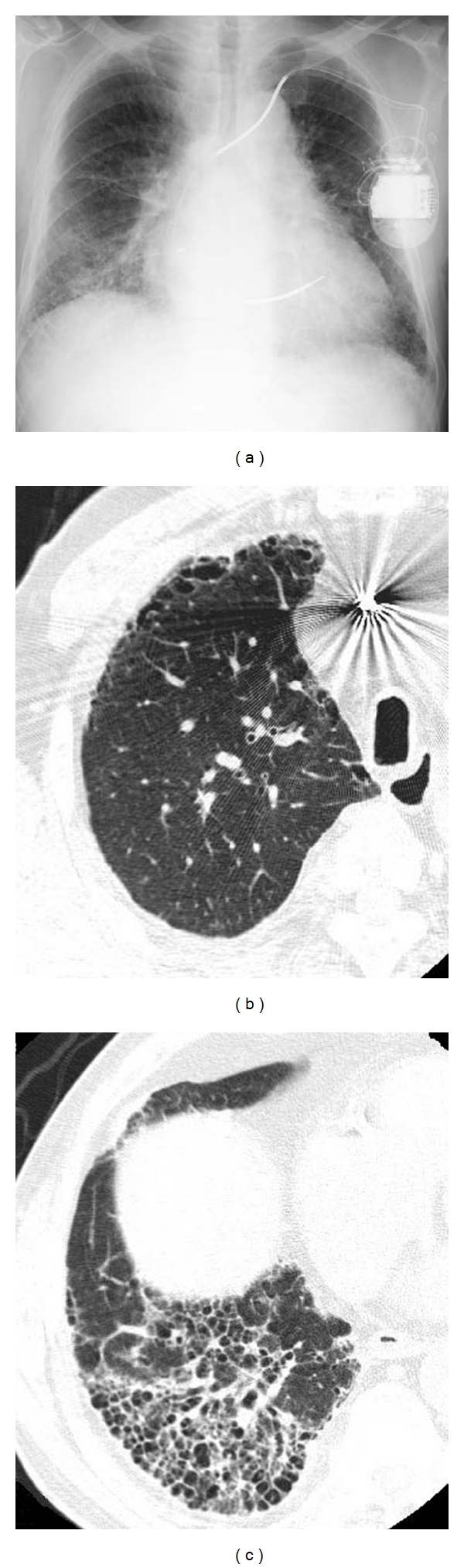
Diffuse interstitial pneumonia (UIP pattern) with emphysema. (a) Chest X-ray shows decreased volume of bilateral lungs. Ground glass opacity including reticular opacity and honeycomb lung in bilateral lower lungs. (b) HRCT of the right upper lobe shows paraseptal emphysema in ventral aspect. (c) HRCT of the right lower lobe shows ground glass opacity and honeycomb lung. Traction bronchiectasis is also observed within the areas of honeycombing.

**Figure 3:**
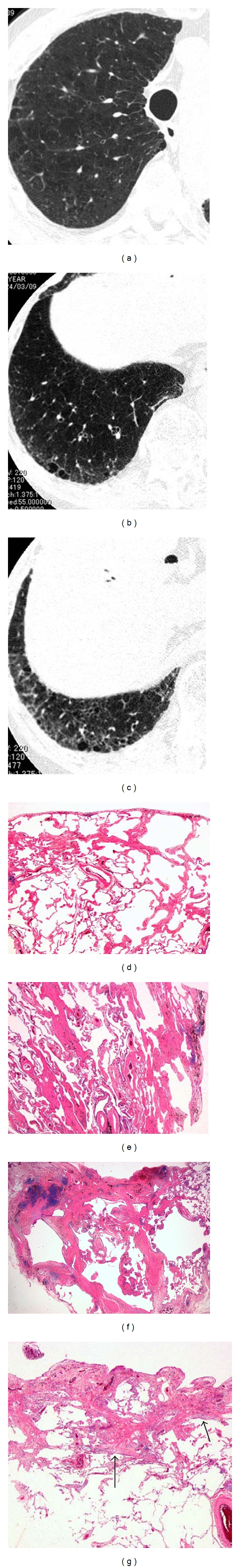
Diffuse interstitial pneumonia (UIP pattern), emphysema with fibrosis. The patient underwent right lower lobectomy for lung cancer of the right lower lobe. (a) HRCT of the right upper lobe shows severe centrilobular emphysema. (b) HRCT of the right lower lobe shows slight ground glass opacity including several thick-walled cyst. (c) HRCT of basilar region shows subpleural ground glass opacity and reticular opacity in the subpleural region: radiologically consistent with UIP pattern. There are some relatively thick-walled cysts in subpleural region; however, no evidence of honeycombing. (d) Pathologic specimen (H&E staining) shows centrilobular and paraseptal emphysema with collagen type fibrosis. (e) Pathologic specimen shows dense centrilobular collagen-type fibrosis, associated with centrilobular emphysema. (f) Pathologic specimen shows cystic lesion in the acinus with destruction of alveoli and dense perilobular and peribronchiolar fibrosis. Normal alveoli adjacent to the cystic lesion. (g) Pathologic specimen shows patchy subpleural and intralobular fibrosis with alternating emphysematous, nonfibrotic area. Note fibroblastic foci (arrows). Sharp border between advanced fibrosis and normally appeared tissue suggests UIP pattern.

Imaging features of CPFE have not been fully described. Radiological findings include centrilobular and/or paraseptal emphysema evident in the upper lungs and IP in the lower lungs [30, 36]. On CXR, volume of lung maintains although there is reticular opacity in bilateral lower lung fields (Figures 1(a), 2(a), and 7(a)). CT patterns of interstitial pneumonia mimic those of IIP in some patients and are unclassifiable in others. Cottin et al. noted that the IP pattern may mimic NSIP (Figure 1), UIP (Figures 2 and 3), OP, RBILD, and other such entities. 

When pulmonary emphysema is present, the radiological pattern of interstitial pneumonia does not necessarily show characteristic one. Akira et al. have described changes in the radiological features of IP in the presence of pulmonary emphysema and noted that the pattern of pathologically proven NSIP with emphysema might mimic the pattern of UIP on HRCT [37].

**Figure 4:**
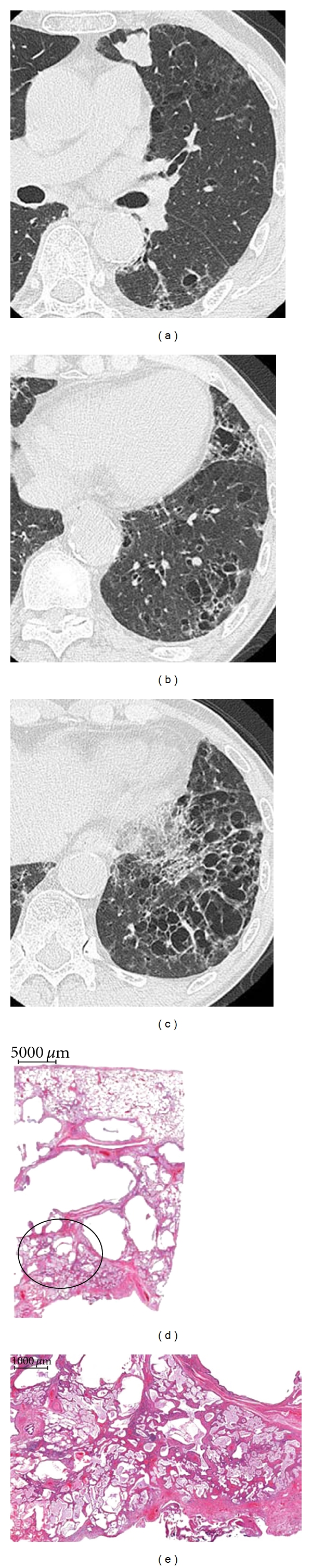
Emphysema with fibrosis, thick-walled large cyst. (a) HRCT of upper lungs shows severe centrilobular emphysema. A relatively thick-walled cyst in the superior basal segment of the left lower lobe. Lung cancer is noted in the lingual division of the left upper lobe. (b) HRCT of left lower lung shows large thick-walled cysts surrounded by ground glass opacity in the regions apart form pleural surface. (c) HRCT of left lower lobe shows multiple bizarre-shaped cysts aggregated in the central portion of the left lower lobe. (d) Pathologic specimens of the resected left upper lobe shows centrilobular cystic lesion involving the membranous and respiratory bronchioles with fibrosis. Note peripheral lung parenchyma spared. (e) Mucus filling in the alveoli with slight septal thickening adjacent to the cyst (circle of Figure 5(e)). Mucus filling and septal thickening correspond to ground glass opacity surrounding large cysts.

Radiological features of CPFE include large relatively thick-walled cysts in addition to pulmonary emphysema (Figure 4). The cysts may be related to large paraseptal emphysema in the upper lungs just beneath the chest wall. Some large cysts may grow within the areas with interstitial pneumonia. These thick-walled cysts probably correspond to emphysema with fibrosis described by Wright et al. [35] or air space enlargement with fibrosis by Kawabata et al. [33], but pathological confirmation as well as determination of the operation of other mechanisms of cyst formation is needed.

As above mentioned, Wright et al. classified smoking-related interstitial changes into two types: emphysema with fibrosis (localized form) and apparent interstitial pneumonia (diffuse form), and HRCT seems to be useful in differential diagnosis of the two forms [35]. However, mixture of the two forms is sometimes observed; apparent interstitial pneumonia pattern with pulmonary emphysema mixed with large thick-wall cysts is sometimes observed (Figures 1, 4, and 6) on HRCT images. Clinical, radiological differences between two forms classified by Wright et al. must be investigated in further study.

**Figure 5:**
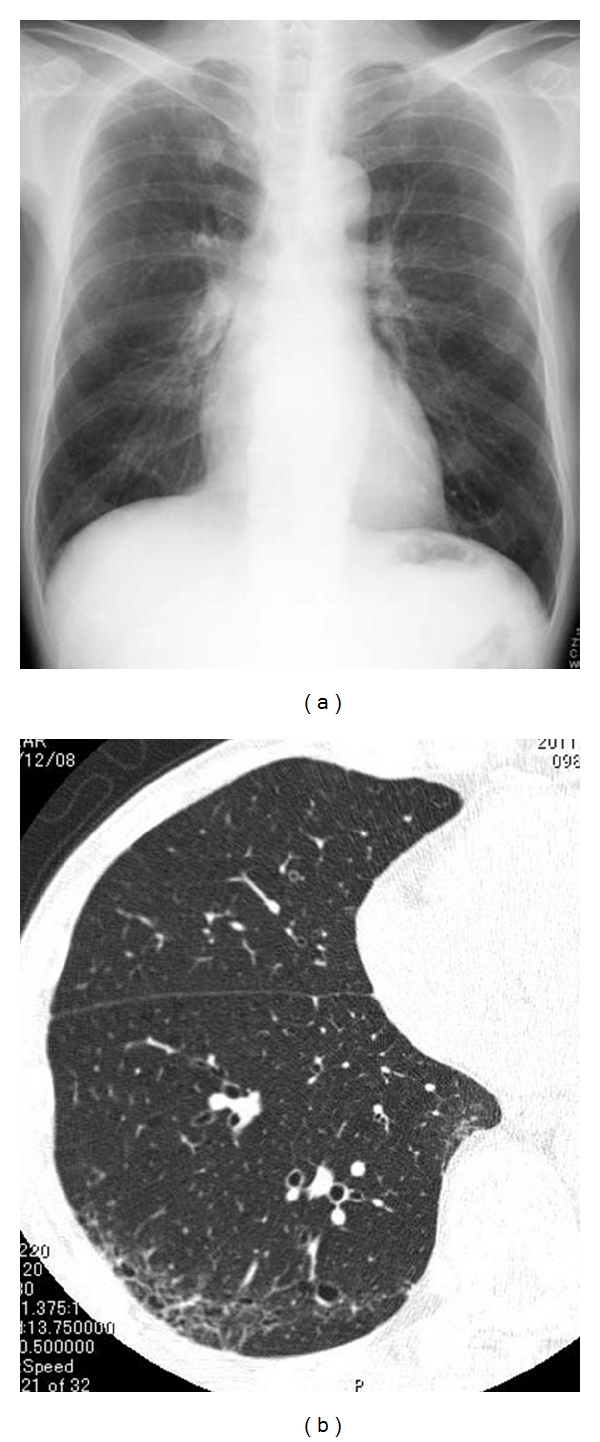
Airway-centered fibrosis with cysts. (a) Chest X-ray shows increased volume of bilateral lungs. (b) High-resolution computed tomography (HRCT) of lower lung shows ground glass opacity and multiple cysts in subpleural region, but these abnormal opacities are predominant in centrilobular region (centrilobular accentuation).

 Other findings include centrilobular relatively thick-walled cyst accompanying interstitial change or centrilobular nodules/subpleural curvilinear opacity. Centrilobular lesions correspond with airway-centered fibrosis, that is, fibrotic changes along the respiratory bronchioles (Figure 5).

 CPFE may show very high prevalence of lung cancer; the possibility of lung cancer must be remained in the radiological differential diagnosis of nodules/mass coinciding with CPFE (Figure 4).

Cottin et al. frequently observed pulmonary hypertension with poor prognosis, but our clinical experience suggests less frequent pulmonary hypertension and relatively good prognosis of interstitial pneumonia [23]. The causes of the difference are unclear but may represent difference in race or disease severity.

**Figure 6:**
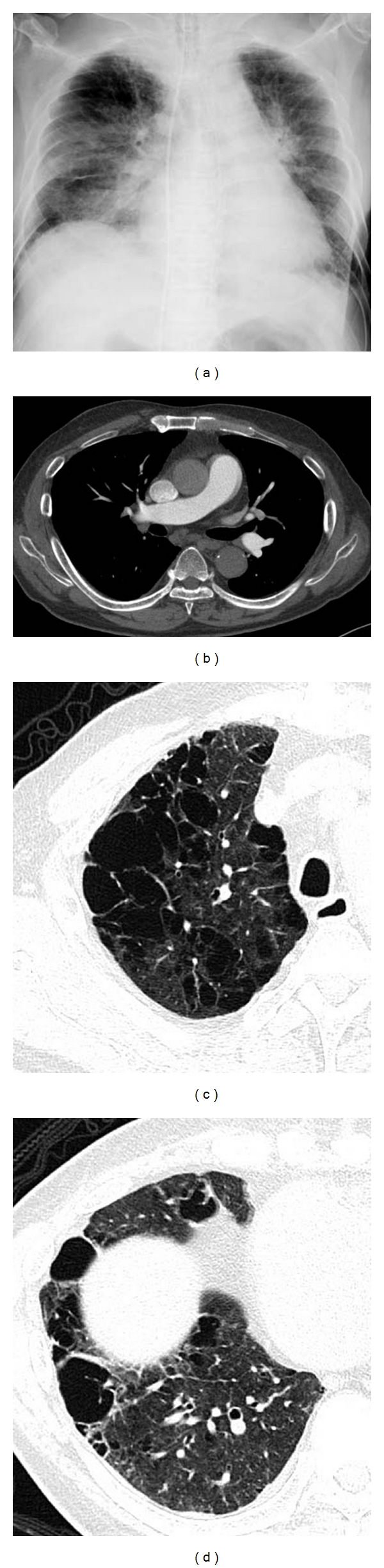
Emphysema with fibrosis, pulmonary hypertension. Estimated right ventricular pressure by cardiac ultrasound is 80 mmHg. (a) Chest X-ray shows cardiomegaly with enlargement of central pulmonary arteries. (b) Contrast-enhanced CT shows dilatation of central pulmonary arteries. There is no evidence of pulmonary thromboembolism. (c) High-resolution computed tomography (HRCT) of upper lung shows severe pulmonary emphysema; some cystic lesions have thick wall. (d) HRCT of the lower lung shows marked destructive change in the lung. There are several large cysts with thick wall.

CT features of pulmonary hypertension are dilatation of the central pulmonary arteries, enlargement of the right-sided heart chambers, reduced number of peripheral pulmonary artery branches, and mosaic attenuation of the lung parenchyma [38] (Figure 6). However, changes in peripheral pulmonary vasculature and parenchyma are unclear when destructive and fibrotic changes in the pulmonary parenchyma are prominent.

**Figure 7:**
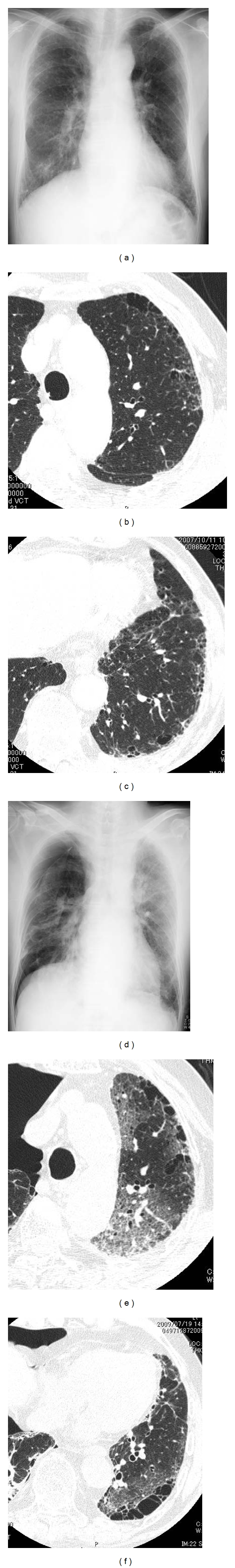
Emphysema with fibrosis, acute exacerbation by anticancer drug. (a)–(e) Imaging findings before acute exacerbation. (a) Chest X-ray shows normal volume of bilateral lungs. There is reticular opacity in bilateral lower lung fields. (b), (c) High-resolution CT (HRCT). In upper lung, centrilobular emphysema, subpleural cyst, and profuse centrilobular small nodular opacities are identified. In lower lung, subpleural cysts and reticular opacity are noted abutting chest wall, most compatible with UIP pattern. (d)–(f) Imaging findings at acute exacerbation. (d) Chest X-ray shows diffuse ground glass opacity in the left entire lung. Pneumothorax is noted on right side. (e), (f) HRCT shows diffuse ground glass opacity overlapping preexisting interstitial shadow.

 Acute exacerbation is less frequent for CPFE than idiopathic pulmonary fibrosis, but its precise frequency has not been determined (Figure 7). Neither is the precise risk of drug-induced lung injury known. Because preexisting IP is a risk factor for drug-induced lung injury, CPFE may affect indication for anticancer drug treatment [39]. In patients with acute lung injury immediately following surgery for lung cancer, acute exacerbation of occult minimal IP might be suspected because this minimal fibrosis is frequently identified in postoperative patients [40].

The radiological features of CPFE correspond to pathologic features of destructive pulmonary changes in smokers, such as emphysema with fibrosis and overt interstitial pneumonia with emphysema. Further investigation is needed to clarify clinical characteristics, complications, and prognosis of CPFE.

## Abbreviations

COP: Cryptogenic organizing pneumonia
COPD: Chronic obstructive pulmonary disease
DIP: Desquamative interstitial pneumonia
GGO: Ground glass opacity
HRCT: High-resolution computed tomography
IIP: Idiopathic interstitial pneumonia
IP: Interstitial pneumonia
IPF: Idiopathic pulmonary fibrosis
LCH: Langerhans cell histiocytosis
NSIP: Nonspecific interstitial pneumonia
RBILD: Respiratory bronchiolitis interstitial lung disease
SRILD: Smoking-related interstitial lung disease
UIP: Usual interstitial pneumonia.
